# Cannabinoids in the Pathophysiology of Skin Inflammation

**DOI:** 10.3390/molecules25030652

**Published:** 2020-02-04

**Authors:** Cristian Scheau, Ioana Anca Badarau, Livia-Gratiela Mihai, Andreea-Elena Scheau, Daniel Octavian Costache, Carolina Constantin, Daniela Calina, Constantin Caruntu, Raluca Simona Costache, Ana Caruntu

**Affiliations:** 1 Department of Physiology, “Carol Davila” University of Medicine and Pharmacy, 050474 Bucharest, Romania; cristian.scheau@umfcd.ro (C.S.); ancab52@yahoo.com (I.A.B.); mihaigratzy@yahoo.com (L.-G.M.); 2 Department of Radiology and Medical Imaging, Fundeni Clinical Institute, 022328 Bucharest, Romania; andreea.ghergus@gmail.com; 3 Department of Dermatology, “Carol Davila” Central Military Emergency Hospital, 010825 Bucharest, Romania; daniel_costache@yahoo.com; 4 Immunology Department, ”Victor Babes” National Institute of Pathology, 050096 Bucharest, Romania; caroconstantin@gmail.com; 5 Department of Pathology, Colentina University Hospital, 020125 Bucharest, Romania; 6 Department of Clinical Pharmacy, University of Medicine and Pharmacy of Craiova, 200349 Craiova, Romania; calinadaniela@gmail.com; 7 Department of Dermatology, “Prof. N. Paulescu” National Institute of Diabetes, Nutrition and Metabolic Diseases, 011233 Bucharest, Romania; 8 Gastroenterology and Internal Medicine Clinic, Carol Davila University Central Emergency Military Hospital, Carol Davila University of Medicine and Pharmacy, 050474 Bucharest, Romania; 9 Department of Oral and Maxillofacial Surgery, “Carol Davila” Central Military Emergency Hospital, 010825 Bucharest, Romania; ana.caruntu@gmail.com; 10 Faculty of Medicine, “Titu Maiorescu” University, 031593 Bucharest, Romania

**Keywords:** cannabinoids, skin cancer, dermatology, inflammation, cell signaling, inflammatory disorders

## Abstract

Cannabinoids are increasingly-used substances in the treatment of chronic pain, some neuropsychiatric disorders and more recently, skin disorders with an inflammatory component. However, various studies cite conflicting results concerning the cellular mechanisms involved, while others suggest that cannabinoids may even exert pro-inflammatory behaviors. This paper aims to detail and clarify the complex workings of cannabinoids in the molecular setting of the main dermatological inflammatory diseases, and their interactions with other substances with emerging applications in the treatment of these conditions. Also, the potential role of cannabinoids as antitumoral drugs is explored in relation to the inflammatory component of skin cancer. In vivo and in vitro studies that employed either phyto-, endo-, or synthetic cannabinoids were considered in this paper. Cannabinoids are regarded with growing interest as eligible drugs in the treatment of skin inflammatory conditions, with potential anticancer effects, and the readiness in monitoring of effects and the facility of topical application may contribute to the growing support of the use of these substances. Despite the promising early results, further controlled human studies are required to establish the definitive role of these products in the pathophysiology of skin inflammation and their usefulness in the clinical setting.

## 1. Introduction

Specific medical benefits of cannabinoids have been unveiled even from ancient times, and the relatively recent discovery of the endocannabinoid system (ECS) has led to a target-based drug discovery approach as emerging research strives to expand the applications of cannabinoids for different diseases, and new cannabinoid molecules are developed to target specific receptors with various affinities [1]. Cannabinoids have been used effectively in different areas of clinical medicine such as the control of nausea, vomiting, and spasticity, the treatment of glaucoma and relief of chronic pain [2]. However, the last decade has been marked by a large number of Phase I and II studies aiming to introduce various cannabinoids as potential treatments in conditions such as Alzheimer’s disease, tuberous sclerosis, epilepsy, glioma, schizophrenia, type 2 diabetes, anxiety disorder, multiple sclerosis, graft-versus-host disease, and many others [3,4,5,6,7]. Their adjuvant or curative potential was also assessed in several neuropsychiatric disorders, but also in oncological and dermatological diseases [8,9,10,11]. The antitumor effects of cannabinoids have been investigated in populational studies, with encouraging results in cancers with rising incidence and prevalence, such as skin melanoma, leukemia, thyroid and liver cancers, diseases that bear high mortality and are encumbering through their complications [12,13,14,15].

New research into the anti-inflammatory properties of cannabinoids has shown mixed but overall positive results [16,17,18,19,20,21,22,23,24,25,26]. The effects of cannabinoids on cancer may also partly arise from their effects on the inflammatory milieu of tumors, and further insight is provided by parallel studies into the pathogenesis of inflammatory and carcinogenic processes and their interferences [27].

Inflammatory skin disorders are a heterogeneous group, implying diverse pathogenic pathways and the involvement of complex regulating signaling loops. Cannabinoids seem to exert their properties on cutaneous inflammation in a dose-dependent manner through receptor-dependent and -independent mechanisms [28,29]. The intricacies of these pathways as well as the molecules involved in the metabolic interferences are addressed in this paper further on.

## 2. Cannabinoids

### 2.1. Description

**Figure 1:**
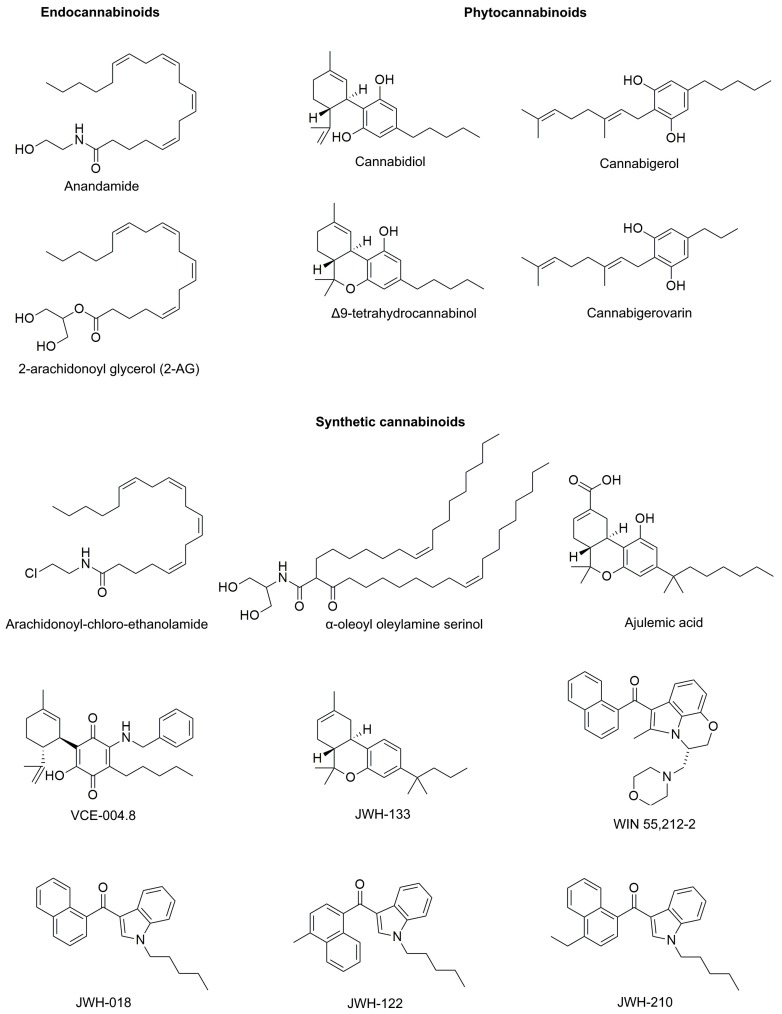
The chemical formulas of the most relevant endo-, phyto-, and synthetic cannabinoids.

“Cannabinoids” is a broad term that includes a large array of substances that share the common property of interacting with cannabinoid receptors (CB). By origin, cannabinoids may be classified either as phyto-, endo-, or synthetic cannabinoids. While only two substances, arachidonoyl ethanolamide (anandamide or AEA) and 2-arachidonoyl glycerol (2-AG), are considered primary endocannabinoids, phytocannabinoids count more than 110 members spanning 11 chemical classes, including psychotropic Δ^9^-tetrahydrocannabinol (THC), while synthetic cannabinoids include hundreds of members divided into 6 classes [30,31,32,33]. The chemical formulas of the most relevant compounds cited in this paper are presented in Figure 1.

Regardless of their source, cannabinoids may elicit their effects on either of the two G protein-coupled cannabinoid receptors, namely CB1 and CB2, an action that can have various efficiency and effects [34].

### 2.2. Receptors

CB1 is widely expressed on the plasma membrane of neurons located in various structures of the central and peripheral nervous systems, more abundantly towards the synaptic terminals [35]. The presynaptic location is related to the role of cannabinoids in modulating neurotransmission, explaining one of the most commonly known effects of anxiolysis, which is accomplished by CB1 mediated decreases of γ-aminobutyric acid and cholecystokinin release [36]. CB1 was identified in various tissues outside of the nervous system, such as the skin, the gastrointestinal tract, the liver, the musculoskeletal system, and various immune cells [37].

CB2 was subsequently discovered, and albeit originally thought of as an exclusively peripheral CB, it was identified in all structures where CB1 was present, including the brain, but with limited expression [38]. Moreover, while the psychoactive properties of cannabinoids appear to be mostly mediated by CB1, CB2 seems to play a more important role in inflammation and pain, and is increasingly recognized as a neuroinflammation biomarker [39].

The existence of non-CB1, non-CB2 G protein-coupled cannabinoid receptors was revealed when endocannabinoids were proven to interact with other molecular targets. Thus, in CB receptors knockout animal models and in in vitro studies on glial cells and vascular endothelium, endocannabinoids induced similar effects [40,41]. Several orphan G-coupled protein receptors (GPR3, GPR6, GPR12, GPR18, GPR55, GPR119, and many others), transient receptor potential channels (TRPV1, TRPV2, TRPV3, TRPV4, TRPM8, and TRPA1), ligand-gated ion channels (5-HT3, glycine, and nicotinic acetylcholine), and peroxisome proliferator-activated receptors (PPARα and PPARγ) were demonstrated to be targeted by, and to some extent mediate the effects of cannabinoids [42,43,44,45].

The normal skin and its appendages are rich in CB1 and CB2 receptors, both identified in keratinocytes, hair follicles, sebaceous glands, melanocytes, fibroblasts, nerve fibers, and adipocytes [46,47,48,49,50,51]. The abundance of cutaneous structures yielding cannabinoid receptors hints to the multiple signaling and regulating functions played by these substances. Benign and malignant skin tumors also express functional CB1 and CB2 receptors, an observation that led to the pursuit of anti-tumoral applications of cannabinoids [52]. Among the non-CB1, non-CB2 receptors targeted by cannabinoids, TRP channels play the most prominent role, as they respond to natural and synthetic cannabinoids that may trigger apoptosis or the release of chemokines and other signaling molecules [46,50,53]. The distribution of TRPs in keratinocytes, hair follicles, mast cells, melanocytes, sebocytes, nerve fibers, and other skin structures facilitates the cannabinoids’ supplementary effects in inflammation and immune response modulation [46,53,54,55,56].

### 2.3. Metabolism

Endocannabinoids are cleaved from membrane phospholipid precursors by distinct synthesis pathways, and are released through a mechanism independent of vesicle secretion [57]. AEA and 2-AG compete for the same facilitated diffusion carrier-mediated transportation and act as partial (AEA) or full (2-AG) agonists on CB1 and CB2 receptors [57,58]. Following uptake, both endocannabinoids are metabolized by fatty acid amide hydrolase (FAAH), cycloxygenase-2, 12- and 15-lipoxygenases, while 2-AG is also hydrolyzed by monoacylglycerol lipase (MAGL) [59,60].

The main phytocannabinoid, THC, has a variable bioavailability depending on the absorption pathway, demonstrating peak plasmatic concentrations in minutes after smoking, one hour after ophthalmic administration and several hours after ingestion [61]. Cannabidiol (CBD), another phytocannabinoid, has a low affinity for CB1 and CB2 and behaves as a negative allosteric modulator of CB1 and CB2 receptors by antagonizing other agonists [62,63]. Also, CBD stimulates the release of AEA by inhibiting FAAH, while also activating various other receptors [64].

Synthetic cannabinoids are usually stronger CB1 and/or CB2 agonists, and due to their large and increasing number of members, they use a multitude of pathways for metabolization, depending on their chemical structure, such as oxidation, hydroxylation, oxidative defluorination, and ester hydrolysis [31].

All cannabinoid classes interfere with the skin cannabinoid receptors and signaling, affecting the homeostasis of skin appendages and cutaneous cells’ metabolisms [65]. FAAH and MAGL have been identified in sebocytes, mast cells, melanocytes, fibroblasts, and other dermal cells, suggesting that the skin is more than an effector of the ECS, acting as a regulatory center of cannabinoids metabolism [50,66,67,68]. The cutaneous ECS is involved in skin differentiation, proliferation, and survival through the actions of AEA and 2-AG that are produced in various skin structures and modulate multiple functions of the skin and its appendages, including hair growth, maintaining the skin barrier integrity, immune response, and the processing of sensory input such as pruritus and pain [69,70,71,72].

Table 1 summarizes cannabinoid types, classes, and their receptor interactions [73,74,75,76,77,78,79,80,81,82,83].

## 3. Inflammation Traits of the Skin

Skin inflammation is a complex, adaptative process, triggered by a multitude of factors, and relying on intricate mechanisms involving cells like platelets, lymphocytes, macrophages, dendritic cells, keratinocytes, and an array of molecules like cytokines, chemokines, and growth factors, interfering with other homeostatic processes such as the immune response, angiogenesis, and apoptosis. Regardless of the initiating factor, inflammatory skin disorders involve increased expression of pro-inflammatory cytokines such as interferons and interleukins, leading to activation of various T helper cells and triggering activation cascades that may not be self-limited, causing the development of a chronic process [84].

The identification of crosstalk between inflammation and cancer in the skin has opened new research directions. Nuclear factor kappa-light-chain-enhancer of activated B cells (NF-κB), a mediator of inflammation, was found to play a pivotal role in activating signaling pathways in skin tumorigenesis [85,86]. Subsequently, numerous in vivo and in vitro studies have found more inflammatory chemokines, cytokines or proteases that are also involved in the transformation, survival, invasion, and metastasis of skin cancer cells, such as tumor necrosis factor-alpha (TNF-α), transforming growth factor-beta (TGF-β), interleukins 6, 17, and 23, matrix metalloproteinases (MMPs) 2 and 9, furin, cathepsin and many more [87,88]. Moreover, inhibition of the inflammatory response yields chemopreventive results, as demonstrated in animal models where the decrease of cyclooxygenase-2 (COX-2) leads to a decline in UVB-induced carcinogenesis [89].

The skin acts as a neuroimmunoendocrine organ with a modulating effect on local blood and lymph flow, sweat glands function, immunoreactivity, and inflammatory response; these effects are facilitated by the integrated nervous system connections that are involved in regulating homeostasis in disruptive conditions such as activation of nociceptors by pain, trauma or inflammation [90,91,92,93,94,95,96]. The concept of dermal neurogenic inflammation was further refined using capsaicin, a molecule that represented the groundstone of inflammation and pain pathogenesis research in the skin, and was subsequently promoted for also having anticarcinogenic and immunomodulatory roles [97,98]. Cannabinoids have proven effective as protective agents in neuroinflammatory disorders, and their anti-inflammatory and immunomodulating effects have also proven beneficial in inflammatory skin disorders [99].

## 4. Cannabinoids’ Role in Inflammatory Skin Disorders

Despite their low rates of mortality, inflammatory skin disorders such as allergic contact dermatitis, psoriasis, acne, scleroderma, and dermatomyositis, have a great impact on the patients’ quality of life and self-esteem. These large array of diseases are associated with a substantial systemic burden of disease and when pruritus and pain are associated, the negative psychological effects are augmented, and the involvement of fibrotic changes may lead to permanent scarring, further enhancing the disease burden [84,100,101,102]. Benefiting from the ease of diagnosing, applying topical treatment and monitoring their evolution due to their superficial location, skin inflammatory disorders represent an increasingly pursued research focus. The development of animal models for these disorders has aided in the investigation of the pathophysiological processes involved, leading to a better understanding of the course of disease and possible mechanisms that may limit, or even revert the development of the illness [103,104,105]. The high impact on the patients’ lives and the healthcare system has prompted the research of new treatments, among which cannabinoids are regarded with growing interest due to their initial favorable results and limited adverse effects. Increasingly more clinical trials are launched with the intent of establishing the effectiveness of cannabinoids in the treatment of skin inflammatory disorders [15].

An added benefit of the usage of cannabinoids in the treatment of inflammatory skin disorders is the possibility of transcutaneous administration. Patches or similar delivery systems can offer a steady and prolonged drug infusion, with minimal local or systemic adverse effects while also avoiding first-pass metabolism [106]. While the absorption of cannabinoids is limited due to their hydrophobic nature, some compounds have higher absorption rates, such as CBD and cannabinol; drug absorption may be further amplified through the use of physical or chemical enhancers [107]. Preclinical and clinical testing on improving the transdermal administration routes is ongoing, aiming to further increase delivery rates and eliminate unwanted side effects [108].

### 4.1. Allergic Contact Dermatitis

Allergic contact dermatitis (ACD) is a type IV delayed hypersensitivity reaction developing as an immune response to an allergen, mediated by T cells and various cytokines and chemokines [109,110].

CBD suppresses the inflammatory reaction of allergic contact dermatitis in vitro, without cytotoxic effects. Thus CBD reduced inflammation in polyinosinic:polycytidylic acid-induced ACD in human keratinocyte (HaCaT) cells, by inhibiting the monocyte chemotactic protein-2 (MCP-2) chemokine and several pro-inflammatory cytokines, such as interleukins (ILs) 6 and 8, and TNF-α [111]. Another in vitro study performed on splenocytes demonstrated similar effects in inflammatory reactions, as CBD decreased the activities of T and B-cells-mediated response, inhibited the release of interleukins 6, 8, and 17, TNF-α, and interferon (IFN)-γ, and modulated the immune response decreasing the activity of T helper 17 cells [112,113,114].

CB1 agonists also demonstrate anti-inflammatory effects in studies using α-oleoyl oleylamine serinol (α-OOS). Therefore, in an in vivo experimental model using phorbol ester-induced acute inflammation developed in mice and atopic dermatitis-like symptoms induced with oxazolone, CB1 agonists proved their anti-inflammatory action through several proposed mechanisms. Mast-cells downregulation, peroxisome-proliferator activated receptors (PPARs) activation, and the decrease of epidermal production of IFN-γ and several chemokines, such as CCL2, CCL8, and CXL10 were the possible anti-inflammatory involved pathways [115,116].

However, conflicting results have been obtained in mice models subjected to CB2 agonists and antagonists by different types of administration. As such, it is considered that CB2 antagonists may decrease inflammation through CB inactivation, but chronic blockade leads to augmenting the inflammatory component of allergic contact dermatitis [116,117,118].

### 4.2. Psoriasis

Psoriasis is an autoimmune inflammatory disorder that may affect the skin, associating epidermal proliferation, due to a dysregulation of the immune system; pro-inflammatory cytokines, including IL-12 and -23 and TNF-α, are produced, and the inflammatory milieu is developed and maintained with contribution from various cells, such as T-helper cells 1 and 17, keratinocytes, and dendritic cells alongside various immune cells [119,120,121,122].

Phytocannabinoids are promising drugs in the treatment of psoriasis due to their effects of inhibiting the proliferation of keratinocytes while also modulating the associated inflammatory component [123]. In human skin cultures, synthetic CB1 agonist arachidonoyl-chloro-ethanolamide (ACEA) inhibited keratinocyte cell proliferation in situ, while decreasing K6 and K16 expression in organ cultured human skin samples [81]. The beneficial effects of phytocannabinoids THC and CBD in psoriasis are the conversion of the pro-inflammatory Th1 profile to an anti-inflammatory Th2 type expression, and the anti-proliferative properties on keratinocytes; however, these effects seem predominantly mediated through PPARγ, independent of CBs, demonstrated by sustained results in CB1 and CB2 blockades on human papilloma virus (HPV)-16 E6/E7 transformed human skin keratinocytes cultures [124].

In 2019, a patent has been filed for the treatment of psoriasis with topical application of cannabidiol and cannabigerol, which showed dose-dependent effectiveness in the trial subjects, apparently via the inhibition of inflammatory cytokines and angiogenic growth factors while restoring the Th1/Th2 balance [125].

### 4.3. Acne

Acne is a skin disease with complex pathogeny, with inflammation at its center, triggered by various processes such as seborrhea, hormonal imbalances, immune reactions, and infectious and environmental factors [126].

Studied performed over 30 years ago have shown that topically applied phytocannabinoids have proven effective in alleviating 12-*O*-tetradecanoylphorbol-13-acetate (TPA)-induced erythema of mouse skin [127]. Moreover, phytocannabinoids safely decrease sebum production, inhibit sebocytes proliferation and reduce the expression of pro-inflammatory cytokines as demonstrated in multiple in vitro and in vivo studies, including a human trial where topical application for 12 weeks showed safety and good results in decreasing erythema and skin sebum [128,129,130]. The positive effects of cannabinoids seem to be non-CB1 non-CB2-mediated, as observed for CBD: CBD inhibits sebum secretion and sebocyte proliferation through TRPV1, 3 and 4 activation, and seems to exert anti-inflammatory effects through A2A adenosine receptors, thus inhibiting the p65 NF-κB pathway [131,132,133]. Conversely, some phytocannabinoids, such as cannabigerol (CBG) and cannabigerovarin (CBGV), trigger an increase in sebum production in human SZ95 sebocytes cell line, possibly due to various affinities for TRP channels and interference with CB receptors [133,134].

A phase 2 trial enrolling over 360 participants and evaluating the effects of a topical cannabinoid named BTX 1503 (a solution made up of 5% CBD as the active ingredient) on acne lesions was recently completed, but the publishing of results is still pending [65,135,136].

### 4.4. Scleroderma

Localized scleroderma is a complex disease featuring inflammation and fibrosis caused by higher than normal collagen deposition; entailing a decrease in functional T regulatory cells, the inflammation in scleroderma shows a specific profile of increased chemokine (C-X-C motif) ligands 9 and 10 (CXCL9 and CXCL10) expression with a decrease in IL-23 and IL-17A T-helper 17-related cytokines [137,138].

Ajulemic acid has proven safe and effective in improving the clinical status of patients with systemic sclerosis in a Phase 2 trial, quantified by the American College of Rheumatology Combined Response Index in diffuse cutaneous systemic sclerosis; the mechanisms were determined to be related to the reduction of inflammation-related genes expression, ascertained on skin biopsies [139]. An international Phase 3 clinical trial of ajulemic acid in scleroderma was initiated in 2018 [140].

The synthetic cannabinoid WIN 55,212-2 has proven effective in impeding the development of skin fibrosis in bleomycin injected DBA/2J mice by preventing fibroblasts activation, as well as inhibiting growth factors expression, such as TGF-β, platelet-derived growth factor-BB (PDGF-BB) and connective tissue growth factor (CTGF) [141].

Another synthetic cannabinoid, VCE-004.8, has also shown benefits in mouse models of scleroderma, reducing vascular collagen deposits, preventing macrophage infiltration, inhibiting the proliferation and migration of fibroblasts and decreasing overall dermal thickness through mechanisms mediated by CB2 and PPARγ; while CB2 seems to mediate the anti-inflammatory effects, such as reducing macrophage IL-1β secretion and reducing the inflammatory infiltrate, PPARγ seems to exhibit anti-fibrotic effects by inhibiting the TGF-β production through interaction with Smads signaling [78,142,143].

### 4.5. Dermatomyositis

Dermatomyositis is an inflammatory myopathy, featuring skin rash and erythema followed by typical muscle tissue necrotic and regenerative processes; with a suspected autoimmune component, the inflammation-driven by activation of lymphocytes and dendritic cells increases the production of interferons and complement factors, which further fuels the destructive environment and may also result in vasculopathy [144].

Ajulemic acid, a synthetic cannabinoid with very high CB2 affinity, triggers the release of endogenous eicosanoids and decreases TNF-α, as well as IFN-α and IFN-β production. In an in vitro study on peripheral blood mononuclear cells isolated from dermatomyositis patients this synthetic cannabinoid reduced pro-inflammatory cytokine secretion [140,145]. Following these initial promising results, ajulemic acid has shown to be a safe, tolerable, and efficient drug in hindering the development of inflammation and fibrosis by promoting pro-resolving versus pro-inflammatory lipid mediators, without inducing immunosuppression. Hence this compound is successfully undergoing phase 2 clinical trials for the treatment of dermatomyositis, when administered orally, as capsules [140,146]. Another randomized controlled trial has reported a reduction in Type 1 and 2 IFN levels and T-helper cell inflammation in patients with dermatomyositis treated orally with ajulemic acid for 12 weeks, compared to those receiving placebo [147]. A phase 3 study for testing the efficacy and safety of ajulemic acid in the treatment of dermatomyositis has been launched in 2019 [148].

## 5. Cannabinoids’ Role in Skin Cancer and Its Associated Inflammation

Skin cancers are a heterogeneous group of diseases, with high prevalence, increasing incidence, potential local and distant complications, and with high mortality rates. Due to their high economic and healthcare impact around the globe, great effort is invested into the research of the physiopathology of these diseases and the development of new and effective treatments [149,150,151,152].

The skin ECS contributes to the regulation of cell differentiation and proliferation through AEA and FAAH that maintain homeostasis through cannabinoid receptor signaling [37,51,124,153]. Both non-melanoma and melanoma skin cancer cells express CB1 and CB2 receptors, alongside other receptors that cannabinoids can activate [154,155]. Cannabinoids have revealed pro-apoptotic and anti-proliferative in various cancers such as prostate, digestive tract and breast carcinomas, with CBD demonstrating the strongest anti-tumor effects [156]. There is a large array of mechanisms that are triggered such as the activation of caspase-3, the increase of Ca2+ concentrations leading to reactive oxygen species (ROS) production stimulation, the induction of apoptosis, reducing the expression of epidermal growth factor (EGF), vascular endothelial growth factor (VEGF), and nerve growth factor (NGF) or their receptors, and inhibiting tumor growth through the AC-cAMP/PKA cascade [157,158]. However, the tumor microenvironment includes an inflammatory component, consisting of leukocytes, cytokines, and various signaling and transcription factors, that aid cancer survival, tumor development, metastasis, and therapy resistance [159]. Among these components, TGF-β can cause immunosuppression and promote tumor growth and survival, TNF-α is involved in cellular transformation, survival, and proliferation, while MMPs can favor tumor invasion through epithelial–mesenchymal transition in many cancers; all these molecules are found in the intersecting pathways of inflammation and carcinogenesis, and cannabinoids can regulate their expression [140,160,161,162,163,164,165,166]. These common anti-inflammatory and anti-carcinogenic effects of cannabinoids make them excellent candidates for cancer treatment, as they act as the immunomodulatory substances that influence cell signaling in the tumor microenvironment.

### 5.1. Melanoma

The deadliest skin cancer with an increasing incidence, melanoma arises from melanocytes mainly subjected to solar or artificial ultraviolet radiation that induces DNA deterioration and enhances immune suppression [167,168].

THC causes autophagy-dependent apoptosis on melanoma models in vivo and in vitro, and the effects are more potent when CBD is associated, inflicting ROS production and caspase activation, suggesting that the two drugs cooperate in inducing apoptosis via different mechanisms [169]. Other authors suggest that the anti-tumoral effects of CBD may also rely on CB2-mediated anti-inflammatory or immuno-modulating activities [170]. CBD alone was tested for anti-tumoral efficacy in melanoma against Cisplatin when administered intraperitoneally on murine B16F10 melanoma tumors, and even though tumor growth restriction and survival length were better for Cisplatin, the quality of life and movement were better in CBD treated animals [171]. Moreover, the in vivo effects of THC on melanoma have been demonstrated to be CB1 and CB2 mediated. This finding was proven in mouse melanoma tumors using cell lines B16 and HCmel12 in wild type and compared to CB1/2^−/−^ mice; furthermore, the suppression of tumor growth on transplanted HCmel12 melanomas in mice was correlated with the antagonistic effects on the tumoral inflammatory milieu [172].

In another melanoma mouse model using B16 melanoma cell lines, synthetic cannabinoids WIN-55,212–2 and JWH-133 decreased tumor cell proliferation via Akt inhibition, causing cell cycle arrest, but with no effects on the MAPK/ERK pathway [155].

Endocannabinoids act similarly on melanoma as they do on non-melanoma skin cancers, promoting tumor death through tumor-toxic AEA metabolites subsequent to COX-2-mediated breakdown, in a dose-dependent and receptor-independent manner [173,174,175].

Conversely, the CB1 receptor was identified as having tumor-promoting effects in knockdown models of melanoma, as the CB1-silenced group demonstrated inhibition of ERK and Akt phosphorylation and cell cycle arrest; these findings lead to the hypothesis that CB1 expression alters communication and feedback loops in the endocannabinoid system, mediating the inhibition of migration and proliferation of melanoma cells in vitro [176]. Furthermore, this controversial pro-tumoral effect observed in vitro may stand to prove that the anti-tumoral effects of cannabinoids cited in various in vivo studies rely on impacting the inflammatory milieu, a factor which is improperly represented in in vitro studies [11,172].

### 5.2. Non-Melanoma Cancers

Squamous cell and basal carcinomas are common malignancies that entail several pitfalls in their treatment such as inadequate skin penetration of chemotherapic drugs and depth-related difficulties in excision [177,178,179,180,181,182]. Systemic application of cannabinoids was proven effective in inducing apoptosis and tumor growth inhibition in vitro and in vivo on PDV.C57 epidermal tumor models. In these models, when using both mixed CB1 and CB2 agonist WIN 55,212-2, but also using CB2 selective agonist JWH-133; the obtained antitumoral effects were the decrease of VEGF, Placental growth factor (PlGF), and Ang2 angiogenic factors expression, impairment of EGF-receptor function with overall reduced blood vessels development and tumoral size regression [52].

Mixed CB1 and CB2 synthetic cannabinoids JWH-018, JWH-122, and JWH-210 demonstrate effective effects in topical application against both carcinogenesis and ear inflammation on a TPA-induced mouse model, hinting at interconnection and interferences between cancer development and inflammation, influenced by CB1 and CB2 [183].

An intrinsic mechanism seems to target non-melanoma skin cancer cells through AEA, which through metabolization by COX-2, becomes an apoptosis-inducing factor; the increase of AEA production appears to amplify the apoptotic results, and the effects are selective towards tumor cells since they express higher levels of COX-2 than surrounding non-tumoral keratinocytes [184,185].

Squamous cell carcinoma may arise under the influence of various biological, physical, or chemical risk factors, such as human papillomavirus infection, ultraviolet radiation exposure, and diverse chemical carcinogens [186,187,188,189,190]. On an ultraviolet B (UVB) irradiation-induced skin carcinogenesis in vivo mouse model, the presence of CB1 and CB2 receptors in wild mice, compared to CB1/2^−/−^ knockdown mice, has correlated with increased tumorigenesis when benzanthracene was added alongside UVB; furthermore, the CB1/2^+/+^ mice had a more prominent inflammation status, with increased TNFα and NF-κB compared to knockdown models, suggesting that CB1 and CB2 receptors are necessary in the pro-inflammatory tumoral response to UVB [191]. However, different cannabinoids may act as either pro- or anti-inflammatory factors, in specific environments and in a dose-dependent manner, due to their complex interferences in the signaling of immune cells [29].

### 5.3. Kaposi Sarcoma

Kaposi sarcoma is a neoplasm caused by Kaposi sarcoma-associated herpesvirus (KSHV), appearing with higher rates in HIV+ patients, with a broad spectrum of morphology; the spindle cells of primary Kaposi sarcoma express basic fibroblast growth factor in high concentrations under the influence of multiple included inflammatory cytokines, such as TNFα, IL-1, and IFN-γ [192,193].

Recent studies have shown mixed results regarding the effects of cannabinoids on Kaposi sarcoma. Synthetic cannabinoid WIN-55,212-2 is effective in vitro, inducing apoptosis on KS-IMM cell lines derived from Kaposi sarcoma, most likely through increased phosphorylation of ERK 1 and 2 triggering subsequent p38 and JNK activation and also noting promotion of caspase 3 and 6 activity [194]. CBD also induces apoptosis in Kaposi sarcoma-associated herpesvirus-infected endothelial cells in vitro by inhibiting viral G protein-coupled receptor (vGPCR) and reducing growth-regulated protein α (GRO-α), VEGF-C and VEGF-receptor 3 levels, thus hindering the tumor cells growth and transformation; GRO-α, a chemokine acting as an agonist for vGPCR, is a key regulator of inflammation, angiogenesis, and tumorigenesis, hinting at an interconnection of these processes in the tumoral environment [195].

Contradictory, another in vitro study showed that THC in low doses acts as a promoter of KSHV, facilitating viral replication through activation of open reading frame 50 (ORF50) protein on primary human dermal microvascular endothelial cells, while also enhancing the viral transmission through increased Platelet endothelial cell adhesion molecule (PECAM)-1 expression [196]. These confounding findings may originate in the generated interferences between the potency of agonists on CB1 and CB2 receptors, respectively, and subsequent signaling pathways involved.

Table 2 highlights the roles of cannabinoids in the inflammation processes associated with various skin disorders.

## 6. Adverse Effects of Cannabinoids

The medical use of cannabis was introduced in the mid-1990s and has been increasingly permitted in the USA and Europe, however, the procedures of prescription are strict and heavily regulated [197]. Due to the large variety of phyto- and synthetic cannabinoids, as well as different concentrations and methods of administration employed, it is difficult to establish a general safety directive.

A natural blend of phytocannabinoids named Sativex, a spray which includes, among others, THC and CBD, has been tested for long term adverse effects, and while almost all users reported some kind of adverse effect, common adverse effects like dizziness and fatigue were reported in more than 10% of patients, mild and serious adverse effects were also noted, including psychiatric events [198].

A meta-analysis including over 1700 patients with chronic neuropathic pain followed over 2 to 26 weeks after treatment with topical, oral, or inhaled phyto- and/or synthetic cannabinoids noted adverse effects such as confusion, dizziness, and sleepiness that led to a higher rate of study drop out compared to placebo, but the evidence is not considered high-quality so further studies are required to establish the confidence of these findings [199].

An extreme complication of inhaled THC is cannabis-induced arterial disease, comprised of two entities, thromboangiitis obliterans and atheromatosis, especially identified in teenagers [200]. IgE-mediated allergic reactions to phytocannabinoids have also been cited, with various severities, and apparently implying cross-allergies with other plant-based foods or beverages [201].

Reports of skin toxicity related to cannabinoids usage are scarce, the most common cutaneous side effects cited being dry skin, urticaria, or pruritus, but the observations were incidental and mostly related to inhalation or ingestion of cannabinoid products, and not due to local effects of transdermal applications [202,203].

The most severe long-term adverse reactions cited for cannabinoids are the pro-tumor effects. THC has been shown to amplify the expression of KSHV GPCR, unleashing the proliferation of endothelial cells and inducing the apparition of Kaposi sarcoma in vitro, on human cell lines; these effects seem to be dose-dependent [196]. Endocannabinoids may be involved in the neural metastasis of melanoma via the CB1 receptor by similarly stimulating tumor cell migration as neuron migration, as demonstrated in vitro on A375 and 501 Mel melanoma cell lines [176,204].

Hindering therapeutic efficiency of human IgG4 monoclonal antibody Nivolumab is an unwanted action. Troubling results have been obtained in an oncologic study featuring the effectiveness of associating cannabis to the human IgG4 monoclonal antibody Nivolumab in the treatment of various cancers including melanoma, as cannabis decreased the response rate to the treatment, hinting at possible interactions between the therapeutic substances [205].

## 7. Summary and Future Perspectives

The term cannabinoid encompasses a large number of substances with different, and sometimes opposite effects on inflammatory processes in the skin. Strong evidence that some compounds have great results in specific conditions is now available. The underlying mechanisms involved in mediating the effects of cannabinoids on various inflammatory conditions, including their implication in the inflammatory milieu of different cutaneous tumors have been discussed in this paper.

Synthetic cannabinoids demonstrate great potential as new and improved formulas are developed and tested. There seems to be a correlation between anti-inflammatory and anti-carcinogenic potency, and as more cannabinoids undergo trials, an enhancement of effectiveness is expected [183].

While policies, regimes and legal limitations partly impede publishing and prescribing medical-cannabis products, the interest in this field is rising as more evidence of the effectiveness of these substances becomes available, and the use of cannabinoids in the treatment of skin disorders may become conventional in the future [206].

## Figures and Tables

**Table 1:** Cannabinoids—structure and ligands.

Cannabinoid	Class	CB1 Affinity/Ki (nM)	CB Receptors Effects	
			CB2 Affinity/Ki (nM)	Efficacy
Anandamide (AEA)	Endo-	89	371	CB1 and CB2 partial agonist (CB1>CB2)
2-arachidonoyl glycerol (2-AG)	Endo-	472	1400	
Cannabidiol (CBD)	Phyto-	4350 ± 390	2860 ± 1230	Non-competitive CB2 antagonist
Δ^9^-tetrahydrocannabinol (THC)	Phyto-	40.7 ± 1.7	36.4 ± 10	CB1 and CB2 partial agonist
Cannabigerol (CBG)	Phyto-	1045 ± 74	1225 ± 85	CB1 and CB2 partial agonist. CB1 competitive antagonist
Cannabigerovarin (CBGV)	Phyto-	-	-	Insignificant effect on CB1 and CB2
Arachidonoyl-chloro-ethanolamide (ACEA)	Synthetic	1.4	> 2000	Selective CB1 agonist
Ajulemic acid (JBT-101)	Synthetic	32.3 ± 3.7	170.5 ± 7.8	CB1 and CB2 partial agonist
α-oleoyl oleylamine serinol (α-OOS)	Synthetic	*unavailable data*	-	Selective CB1 agonist
WIN 55,212-2	Synthetic	1.89 ± 0.09	0.28 ± 0.16	CB1 and CB2 full agonist
VCE-004.8	Synthetic	> 40,000	170 ± 50	Selective CB2 agonist
JWH-133	Synthetic	677 ± 132	3.4 ± 1	Selective CB2 full agonist
JWH-018	Synthetic	9.00 ± 5.00	2.94 ± 2.65	CB1 and CB2 full agonist
JWH-122	Synthetic	0.69 ± 0.05	1.2 ± 1.2	CB1 and CB2 full agonist
JWH-210	Synthetic	1.43 ± 0.39	0.94 ± 0.19	CB1 and CB2 full agonist

**Table 2:** Summary of the roles of cannabinoids in the inflammation associated with various skin disorders.

Disease	Cannabinoid	Direct anti-Inflammatory Effects	Indirect anti-Inflammatory /Other Effects	Model	Reference
Allergic contact dermatitis	CBD	Inhibition of MCP-2, IL-6, IL-8 and TNF-α	-	HaCaT cells (in vitro)	[111]
	CBD	Inhibition of IL-6, IL-8, IL-17, TNF-α, and IFN-γ	Inhibition of T-cells and B-cells mediated response	Splenocytes (in vitro)	[112]
	α-OOS	PPARs activation, decrease of IFN-γ, CCL2, CCL8 and CXL10	Mast-cells downregulation	Oxazolone mouse model (in vivo)	[115]
Psoriasis	ACEA	-	Inhibition of keratinocyte cell proliferation in situ; decrease of K6 and K16 expression		[81]
	THC and CBD	-	Inhibition of keratinocyte cell proliferation	HPV-16 E6/E7 transformed human skin keratinocytes cultures (in vitro)	[124]
Acne	THC and CBD	-	Inhibition of cyclooxygenase and lipoxygenase	TPA-induced erythema in mice (in vivo)	[127]
	Mixture (Cannabis seeds extract)	Decrease of erythema	Decrease of sebum production	Human volunteers (*trial*)	[128]
	CBD	Inhibition of the pro-inflammatory p65 NF-κB pathway	-	SZ95 human sebocytes culture (in vitro)	[133]
Scleroderma	Ajulemic acid	Reduction of inflammation-related genes expression	-	Patients with systemic sclerosis (*trial*)	[139]
	WIN 55,212-2	Inhibition of expression of TGF-β, PDGF-BB and CTGF	Prevention of fibroblasts activation	Bleomycin injected DBA/2J mice (in vivo)	[141]
	VCE-004.8	Reduction of IL-1β secretion, inhibition of TGF-β production	Reduction of macrophage infiltration	Bleomycin-induced dermal fibrosis murine model (in vivo)	[78]
Dermatomyositis	Ajulemic acid	Release of endogenous eicosanoids and decrease of TNF-α, IFN-α and IFN-β production	-	Peripheral blood mononuclear cells isolated from dermatomyositis patients (in vitro)	[145]
	Ajulemic acid	Increased production of pro-resolving vs pro-inflammatory lipid mediators	-	Patients with diffuse cutaneous systemic sclerosis (*trial*)	[146]
	Ajulemic acid	Reduction of Type 1 and 2 interferon levels as well as T-helper cell inflammation	-	Patients with skin-predominant dermatomyositis (*trial*)	[147]
Melanoma	THC and CBD(Sativex)	ROS production and caspase activation through undetermined mechanism (possibly implying anti-inflammatory effects of CBD)	-	Mice bearing BRAF wild-type melanoma xenografts (in vivo)	[169]
Non-melanoma skin cancer	Undetermined	Decrease of TNFα and NF-κB	-	UVB-induced skin carcinogenesis mouse model (in vivo)	[191]
Kaposi sarcoma	CBD	Reduction of GRO-α	Inhibition of vGPCR and reduction of VEGF-C and VEGFR-3	Kaposi sarcoma–associated herpesvirus-infected endothelial cells (in vitro)	[195]
